# New insights into *Phakopsora pachyrhizi* infection based on transcriptome analysis *in planta*

**DOI:** 10.1590/1678-4685-GMB-2017-0161

**Published:** 2018

**Authors:** Michelle Pires Rincão, Mayra Costa da Cruz Gallo de Carvalho, Leandro Costa Nascimento, Valéria S. Lopes-Caitar, Kenia de Carvalho, Luana M. Darben, Alessandra Yokoyama, Marcelo Falsarella Carazzolle, Ricardo Vilela Abdelnoor, Francismar Correa Marcelino-Guimarães

**Affiliations:** ^1^ Programa de Pós-Graduação em Genétiva e Biologia Molecular, Departamento de Biologia Geral, Universidade Estadual de Londrina, Londrina, PR, Brazil; ^2^ Laboratory of Plant Biotechnology and Bioinformatics, Embrapa Soja, Londrina, PR, Brazil; ^3^ Universidade Estadual do Norte do Paraná, Bandeirantes, PR, Brazil; ^4^ Laboratory of Genomics and Expression (LGE), Instituto de Biologia, Universidade Estadual de Campinas (Unicamp), Campinas, SP, Brazil; ^5^ Programa de Pós-Graduação em Biotecnologia, Universidade Estadual de Londrina, Londrina, PR, Brazil

**Keywords:** Asian soybean rust, multigenic families, transposable elements

## Abstract

Asian soybean rust (ASR) is one of the most destructive diseases affecting soybeans. The causative agent of ASR, the fungus *Phakopsora pachyrhizi*, presents characteristics that make it difficult to study *in vitro*, limiting our knowledge of plant-pathogen dynamics. Therefore, this work used leaf lesion laser microdissection associated with deep sequencing to determine the pathogen transcriptome during compatible and incompatible interactions with soybean. The 36,350 generated unisequences provided an overview of the main genes and biological pathways that were active in the fungus during the infection cycle. We also identified the most expressed transcripts, including sequences similar to other fungal virulence and signaling proteins. Enriched *P. pachyrhizi* transcripts in the resistant (PI561356) soybean genotype were related to extracellular matrix organization and metabolic signaling pathways and, among infection structures, in amino acid metabolism and intracellular transport. Unisequences were further grouped into gene families along predicted sequences from 15 other fungi and oomycetes, including rust fungi, allowing the identification of conserved multigenic families, as well as being specific to *P. pachyrhizi*. The results revealed important biological processes observed in *P. pachyrhizi*, contributing with information related to fungal biology and, consequently, a better understanding of ASR.

## Introduction

The plant pathogenic basidiomycete fungus *Phakopsora pachyrhizi* (Sydow & P. Sydow) causes the disease known as Asian soybean rust (ASR). ASR is one of the diseases that causes the most significant losses in soybean (*Glycine max*(L.) Merrill) crops and is of great concern because it is a polycyclic disease with high destructive power (Scherm *et al.*, 2009). *P. pachyrhizi* reproduction is predominantly, if not exclusively, asexual (Anderson *et al.*, 2008). Once in contact with a leaf surface, the urediniospores, an asexual form the spores, germinate to initiate the rapidly progressing infection process, and new urediniospores are formed and released within five to eight days through sporulation structures (uredinias), initiating a new cycle of infection (Zambolin, 2006; Morales *et al.*, 2012). As an obligatory biotrophic organism, the development of *P. pachyrhizi* occurs only in living tissue, hampering the study of fungal biology (Voegele *et al.*, 2009).

The infection strategies used by biotrophic organisms have received increased attention in the last decade as a result of an increasing availability of genomic data for these pathogens, such as the sequencing of the genomes of *Blumeria graminis*, *Puccinia graminis* f.sp *tritici*, *Melampsora larici-populina, Puccinia striiformis* f.sp. *tritici*, and *Melampsora lini* (Spanu *et al.*, 2010; Cantu *et al.*, 2011; Duplessis *et al.*, 2011; Nemri *et al.*, 2014). These data allowed the identification of some adaptive characteristics that have been preserved throughout the evolutionary process, such as those related to adaptation to an extreme parasitic lifestyle by the loss of nitrate and sulfate assimilation pathways; in contrast, there has been an expansion of gene families related to pathogen nutrient acquisition and effector delivery by the haustorium (Cantu *et al.*, 2011; Duplessis *et al.*, 2011; Nemri *et al.*, 2014).

Since the nuclear genome of *P. pachyrhizi* has not yet been sequenced, most of the molecular information about this pathogen has been obtained from work involving transcriptome sequencing associated with bioinformatics analyses. Thus, a moderate repertoire of candidate gene sequences expressed in germinated urediniospores, appressorium, haustorium, and uredinia have become available (Posada-Buitrago and Frederick, 2005; Tremblay *et al.*, 2009, 2012, 2013; Stone *et al.*, 2012; Link *et al.*, 2014; Carvalho *et al.*, 2016). Most of these studies focused on the characterization of specific molecular mechanisms during the infection process, with a concentration on analyses of specific structures of the pathogen, such as the urediniospores and haustorium, or of the total infected leaf. To date, studies focused on characterizing the *P. pachyrhizi* transcriptome by associating high-performance sequencing, and sample enrichment with pathogen tissues in compatible and incompatible interactions via LCM (laser capture microdissection) (Emmert-Buck *et al.*, 1996) of the lesion are still rare.

This work enabled the characterization of the *in planta* transcriptome of *P. pachyrhizi*, allowing an overview of the obtained transcripts and the molecular processes that occur at 10 days post-infection, as well as the identification of most expressed sequences and enriched pathogen transcripts present in the resistant genotype PI561356 and among different infection structures. Comparative analyses between the transcripts of *P. pachyrhizi* and other fungi allowed grouping transcripts in multigenic families common to different species of fungi, including other rust species and *P. pachyrhizi*, revealing families that were conserved between the different analyzed species and specific to *P. pachyrhizi*. The prediction of active transposable elements allowed the identification of different subclasses of retrotransposons and DNA transposons elements in the *P. pachyrhizi* transcriptome. Finally, RT-qPCR analysis validated the expression levels of *P. pachyrhizi* genes based on the deep sequencing results.

## Materials and Methods

### *P. pachyrhizi* transcriptome during host interactions

#### Experimental design and inoculation

The experiment was completely randomized, with three replicates per treatment and three plants per pot, using genotypes PI561356, which has an *R* gene that maps close to the *Rpp1* gene (Kim *et al.*, 2012), and the susceptible soybean BRS231 (Ribeiro *et al.*, 2007). The plants were grown in a greenhouse under controlled conditions of temperature and humidity until they reached developmental stage V2 (Fehr *et al.*, 1971), when they were inoculated with the fungus. The inoculum used in this work was obtained from a Brazilian population of *P. pachyrhizi*. Urediniospores were propagated for more than 10 generations in the susceptible genotype BRSMS-Bacuri under controlled greenhouse conditions. For inoculation, urediniospores were collected and resuspended in solution containing 0.05% Tween20 (v/v) to a final concentration of 1.3 x 10^5^ spores per mL. The same solution without spores, however, was used for mock-inoculated leaves (mock plants) as a control for the inoculation procedure. The plants were covered with plastic bags for two days to optimize pathogen infection of the plant and to avoid cross-contamination of the control, mock-inoculated plants. At 10 days post-inoculation (dpi), we observed TAN (BRS 231) and RB (PI561356) lesions on the underside of the leaf of the inoculated plants, but not on the mock-inoculated controls. At this time, pieces of leaves containing rust lesions were collected for the laser capture microdissection (LCM) procedures.

#### Laser capture microdissection (LCM)

Leaf segments of 1 cm^2^ with lesions were randomly sampled from the third trifoliate of infected plants and immediately fixed on ice in Farmer’s solution containing 75% ethanol (v/v) and 25% acetic acid (v/v) (Kerk *et al.*, 2003). Pieces of leaves were fixed overnight at 4 °C in Farmer’s solution. On the second day, the Farmer’s solution was removed, and the leaf samples were washed twice with 75% (v/v) cold ethanol and dehydrated using a series of graded ethanol:xylene solutions. The samples were embedded in paraffin at 58 °C and transferred into xylol:paraffin solutions, in which the paraffin concentration was increased with each transfer (every four hours), culminating in the transfer of samples to pure paraffin in the fourth and final transfer, as described by Cai and Lashbrook (2006). All paraffin-embedded blocks were stored for three weeks at -20 °C until LCM. Immediately before the LCM procedure, 12-μm sections were cut using a rotating microtome and transferred to membrane microscope slides. The sections were dewaxed in a series of xylol:ethanol solutions, dehydrated in graded ethanol series, and stained with fast green acid and fuchsine. Twenty sections containing a variable number of rust lesions were prepared for each biological replicate. For this study, the PixCell II LCM system (Arcturus, Sunnyvale, CA, USA) and CaPSURE Macro LCM (Arcturus) were used to collect fungal and leaf cells. Each cap was used for pulses ranging from 1500–2000 corresponding to the collection of approximately the same number of cells. Samples were collected for both resistant and susceptible plants (Carvalho *et al.*, 2016).

#### RNA isolation and sequencing

RNA was extracted from cells collected separately from each biological replicate, so the RNA extracted from 4,500 to 6,000 cells represents each genotype. Total RNA (< 10 ng) was extracted from mesophilic cells collected from rust infection sites using the PicoPure RNA isolation kit (Arcturus). Two rounds of RNA amplification were performed with RiboAmp HS Plus (Arcturus) to obtain a final yield of approximately 18 μg of amplified RNA (aRNA). The quality and quantity of the aRNA were determined using a 2100 Bioanalyzer (Agilent, Palo Alto, CA, USA). cDNA synthesis was performed before sequencing using the First Strand Super Script III kit (Invitrogen) following the manufacturer’s recommendations. The high-performance paired-end (54 bp, 250 bp insert size) and single-end (100 bp) sequencing were obtained using the Illumina Genome Analyzer GAiix platform (San Diego, CA, USA).

#### Construction of the P. pachyrhizi transcriptome

Initially, low-quality sequences were removed (Phred score < 20) and the remaining RNA-Seq reads were aligned with the soybean reference genome Williams 82 (Schmutz *et al.*, 2010) using the TopHat software (Trapnell *et al.*, 2009). Reads without alignments were assembled *de novo* using the Trinity assembler (Grabherr *et al.*, 2011). Two different assemblies were performed as follows: (i) using only single-end reads (minimum contig size of 200 bp), and (ii) using only paired-end reads (minimum contig size of 100 bp). To increase the set of transcript reference data, the Sanger sequencing *P. pachyrhizi* ESTs available at the National Center for Biotechnology Information (NCBI) were assembled into contigs and singlets. The ESTs were adjusted using the bdtrimmer software (Baudet and Dias, 2005) to discard low quality, vector, and short sequences, and possible soybean contamination was estimated using BLASTN (Altschul *et al.*, 1997) against the soybean genome. The remaining sequences were assembled using CAP3 (Huang and Madan, 1999) to produce 6,105 sequences. The two assemblies were merged using CAP3 with a minimum overlap of 40 bp and minimum identity of 90%. Finally, Sspace (Boetzer *et al.*, 2011) was used to scaffold the CAP3 result, producing 53,405 scaffolds (mean size, 433.1 bp). All 53,405 sequences from the *de novo* assembly were aligned with the soybean genome (BLASTN, e-value cutoff of 1e-10), with the GenBank nr database (BLASTX, e-value cutoff of 1e-5) and with two local fungal databases: (i) 78,105 proteins from the following plant-interacting fungus: *L. bicolor*, *P. graminis* f. sp. *tritici*, *U. maydis*, *M. larici-populina*, and *M. oryzae*; and (ii) 840,789 *P. pachyrhizi* genomic sequences (accession numbers 451441523–455661682). Sequences that aligned with the soybean genome and not with our local fungal database were considered soybean contigs. Sequences that showed a high similarity to plant sequences and did not align with the soybean genome or with fungal sequences were also considered to be from soybean. Sequences with a positive match in soybean, but with high similarity to the fungal databases were considered to be *P. pachyrhizi* contigs. All other sequences were considered to be *P. pachyrhizi* contigs. The final *P. pachyrhizi* dataset consisted of 36,350 contigs. All reads used in the *de novo* assembly step were mapped onto the contigs using SOAP2 (Li *et al.*, 2009), and the alignment result was used to calculate the expression level of each contig by FPKM (fragments per kilobase million) (Mortazavi *et al.*, 2008). All generated sequences, from soybean and from *P. pachyrhizi*, are available in the *P. pachyrhizi* transcriptome LGE database (http://bioinfo03.ibi.unicamp.br/phakopsora/).

*P. pachyrhizi* transcripts were aligned and annotated against the GenBank non-redundant (NR) protein database (BLASTX, e-value cut-off of 1e-5), GenBank nucleotide database (NT) (BLASTN, e-value cutoff of 1e-5), conserved domains database (CDD) (Marchler-Bauer *et al.*, 2011) (RPSBLAST, e-value cut-off of 1e-3), the reference soybean genome Williams82 (BLASTN, e-value cutoff of 1e-10), and finally against the local fungal databases. Functional annotations for the *P. pachyrhizi* transcripts were performed using the Blast2GO software (Conesa *et al.*, 2005), refining the annotations using the Annex and GO-Slim mapping functions for yeast and InterProScan, and merging the results with the functional annotations. Additionally, the Enzyme Code and KEGG (Kyoto Encyclopedia of Genes and Genomes) functions were used for the identification of metabolic pathways and enzymatic codes.

#### Enriched categories in the P. pachyrhizi transcriptome

Enrichment analysis (Fisher’s exact test – FDR adjusted *p*-value < 0.05) available in Blast2GO was performed to identify GO terms that were overrepresented in each genotype, susceptible and resistant (BRS231 and PI561356, respectively), and GO terms that were overrepresented and conserved among other *P. pachyrhizi* infection structures. To identify enriched GO terms among genotypes, we used contigs with FPKM values equal to or greater than 1 for each of the genotypes. To identify GO terms that were common to different infection structures, a total of 1,029 ESTs from germinated urediniospores and appressoria (Stone *et al.*, 2013) and 4,483 ESTs from haustoria (Link *et al.*, 2014) were separately aligned against the 36,350 *P. pachyrhizi* unisequences (leaf lesion) using WU-BLAST (Washington University BLAST - http://blast.wustl.edu). The sequence results (only the sequences that presented a similarity greater than or equal to 90%) were then used in the Venn analysis. The sequences common to infection structures were then subjected to enrichment analysis using Blast2GO software.

#### Comparative analysis

To identify multigenic families that were conserved between the *P. pachyrhizi* transcriptome and other fungal genomes and transcriptomes, we performed a clustering analysis using OrthoMCL software (Li *et al.*, 2003). Multigenic family construction was performed using sets of proteins predicted from publicly available genomes: 12 fungal genomes [10 Basidiomycetes – *M. larici-populina* (Joint Genome Institute - JGI), *P. graminis* f. sp. *tritici* (Broad Institute), *Coprinopsis cinerea* (Broad Institute), *Cryptococcus neoformans* (Broad Institute), *Postia placenta* (JGI), *Laccaria bicolor* (JGI), *Malassezia globosa* (JGI), *Phanerochaete chrysosporium* (JGI), *Sporobolomyces roseus* (JGI, v1) and *Ustilago maydis* (Broad Institute); two Ascomycetes - *Neurospora crassa* (Broad Institute) and *Magnaporthe grisea* (Broad Institute)]; and two oomycetes genomes [*Phytophthora sojae* (JGI) and *Phytophthora infestans* (Broad Institute)]. In addition to the previously sequenced genomes listed above, we also used the EST datasets available for the rusts *Uromyces appendiculatus* and *P. pachyrhizi* (Link *et al.*, 2014)*.* These sets of sequences were combined with the transcriptome results obtained herein for *P. pachyrhizi*. All data were compiled into a single FASTA file that was used in the independent version of the software OrthoMCL version 1.4 (http://orthomcl.org/common/downloads/software/unsupported/v1.4/) with a MCL standard and inflation parameter of 1.5. Thus, based on the similarity of the protein sequences, they were grouped into OrthoMCL multigenic families.

A comparative analysis was performed using three parameters: a) families common to all 16 species used; b) families common to basidiomycetes (covering the species *M. larici-populina*, *P. graminis*, *C. cinerea*, *C. neoformans*, *P. placenta*, *L. bicolor*, *M. globosa*, *P. chrysosporium*, *S. roseus, U. maydis, U. appendiculatus*, and *P. pachyrhizi*); and c) families common to rust fungi (Pucciniales) (covering the species *M. larici-populina*, *P. graminis*, *U. appendiculatus*, and *P. pachyrhizi*). Finally, we also identified *P. pachyrhizi* exclusive families.

#### Prediction of transcriptionally active transposable elements

*P. pachyrhizi* unisequences were compared against the transposable element (TE) sequences available in the Repbase protein transposable element database (Kapitonov and Jurka, 2008) using the computer RepeatMasker tool open version 4.0.6. The query species was assumed to be the fungal dataset available in the Repbase database. Unigenes were considered related to TEs according to the RepeatMasker parameters “selective and matches to coding sequences” available at http://www.repeatmasker.org/webrepeatmaskerhelp.html.

#### Validation of RNA-Seq expression levels by RT-qPCR

Quantitative PCR (RT-qPCR) was performed using samples of spores and germinated spores (*in vitro* growth) and infected leaf tissue (*in vivo* growth). Spores (S) and germinated spores (GS) were obtained from the fresh spores of *P. pachyrhizi* cultivated on detached soybean leaves and maintained in Petri dishes under controlled temperature and humidity conditions in a heated chamber. Germinated spores were obtained from fresh spores deposited in a Petri dish containing water solution with 0.04% Tween and incubated overnight for 16 hours. Infected leaf tissue was collected from the susceptible soybean genotype (Williams82) maintained in a greenhouse for fungal inoculation and subsequent sample collection. Inoculation was performed at stage V2 of development on the second foliolate using the Brazilian *P. pachyrhizi* isolate LD5511 (shows virulence in *Rpp1*, *Rpp2, Rpp3*, and *Rpp5* soybean genotypes) (Darben LM, 2013, Doctoral Thesis, Universidade Estadual de Maringá, Maringá, Brazil) at a concentration of 1 x 10^5^ spores/mL. Infected leaves of compatible interactions were collected at different times after inoculation (0, 6, 12, 24, 48, 72, 96, and 192 hours post-inoculation “hpi”) to represent the progression of infection and host tissue colonization by the fungus. Once harvested, fungus and plant were immediately frozen in liquid nitrogen and stored at -80 °C. The experiment consisted of three biological replicates containing three plants each.

Total RNA was extracted from 100 mg of frozen leaf tissue samples and 30 mg spores and germinated spores using the RNeasy Plant Mini Kit (Qiagen). RNA contamination with genomic DNA was eliminated by treatment of 1 μg of RNA with RNase-free DNase (Invitrogen). The cDNA was synthesized using the First Strand Super Script III kit (Invitrogen) following the manufacturer’s recommendations. RT-qPCR was performed using the Real Time StepOnePlusTM equipment (Applied Biosystems) with SYBR green for detection of double-stranded PCR products. Primers were designed using Primer3Plus software based on the sequences of the six *P. pachyrhizi* transcripts (Table S1). All primers were first tested by standard PCR using DNA and soybean cDNA to ensure specific amplification for *P. pachyrhizi*. Three negative controls were used to ensure that only the cDNA of *P. pachyrhizi* was amplified. The efficiency of the primers was calculated based on the equation [10^(-1/slope)^] - 1 (Pfaffl, 2001). Each PCR reaction was performed in triplicate, and the specificity of the amplification products was validated by analyzing the dissociation curve. Expression levels were determined by the 2^-^ΔCt method. The endogenous fungal tubulin gene was used for normalization (Maciel *et al.*, 2010). RT-qPCR expression levels obtained were used to support the deep sequencing results, and thus the FPKM values obtained through RNA-Seq data were also normalized to the FPKM values of the endogenous tubulin gene.

## Results

### Transcriptome overview

The transcriptome of *P. pachyrhizi* on infected soybean leaves was obtained by inoculating a resistant genotype (PI561356 - *Rpp1b*) and a susceptible genotype (BRS231) with a *P. pachyrhizi* isolate that was maintained in a greenhouse. At 10 dpi, sections of leaf containing RB (PI561356) or TAN (BRS 231) lesions were collected, and the tissues were fixed and prepared for LCM. The LCM methodology was used to enrich the transcripts of the fungus in relation to the plant transcripts, dissecting fungal cells and adjacent cells of the mesophyll. RNAs present in the LCM samples were identified by Illumina sequencing. A total of 92 million reads of 100-bp single-end and 54-bp paired-end (insert size of 250 bp) were generated for the susceptible (BRS231) and resistant (PI561356) soybean genotypes.

A total of 144,659 contigs were generated after the removal of low-quality reads and reads that aligned with the soybean genome (*Glycine max Wm82.a2.v1*) or soybean predicted transcripts (Schmutz *et al.*, 2010). The remaining reads were used for the *ab initio* assembly of *in plant P. pachyrhizi* transcripts at 10 dpi. To improve the assembly quality, *P. pachyrhizi* ESTs from Sanger sequencing reads available from NCBI were edited and assembled into 6,105 contigs (with a mean size of 625 bp). The three assemblies were merged to generate a total of 53,405 contigs comprising 17,055 from soybean sequences and 36,350 unique *P. pachyrhizi* sequences (unisequences) expressed at 10 dpi *in plant*.

The combined assembly resulted in an increase in the size of the contigs and a higher percentage of contigs that were mapped to the local database of fungal sequences. A total of 11,614 (31.95%) transcripts from *P. pachyrhizi* showed similarity to proteins encoded by other phytopathogenic fungi, especially the rust fungi *P. graminis* (9,979 sequences) and *M. larici-populina* (9,362 sequences), for which the complete genomes are available (Duplessis *et al.*, 2011). It is important to note that 64.07% of the sequences (23,290 sequences) exhibited similarity to genomic reads of *P. pachyrhizi* (Table 1).

**Table 1:** General statistics of the *de novo* assembly of RNA-Seq data.

Parameters	*in planta* Pp transcriptome
Number of contigs	36,350
Average contigs size (bp)	471.68
Size of largest contig (bp)	7,874
Size of smallest contig (bp)	100
Average number of reads per contig	2,077.11
Number of annotated contigs^1^	
Against NR	19,573
Against NT	12,520
Against CDD	11,626
Against local fungi database^2^	11,614
Against *P. pachyrhizi* genomic reads	23,290
Against soybean genome	106
Blast2GO general results	
Total GO terms	14,043
Contigs with functional annotation	5,622
Contigs with identified domains	4,187
Contigs in metabolic pathways	1,519
Contigs with enzymatic codes	1,129

1 Number of annotated contigs: number of contigs aligned in NR (BLASTX, e-value cutoff 1e-5), NT (BLASTN, e-value cutoff 1e-5), Conserved Domains Databases (CDD) (BLASTX, e-value cutoff 1e-5), local fungi database (BLASTX, e-value cutoff 1e-5), *P. pachyrhizi* genomic reads and soybean genome (BLASTN, e-value cutoff 1e-5).

2 Local fungi database: 78,105 proteins of five phytopathogenic fungi – 23,132 of *L. bicolor* (JGI, v. 2.0), 20,566 of *P. graminis* (Broad Institute), 6,522 of *U. maydis* (Broad Institute), 16,831 of *M. larici-populina* (JGI, v. 1.0), 11,054 of *M. grisea* (Broad Institute), and 840,789 genomic reads of *P. pachyrhizi* (NCBI).

Functional annotation was performed using Blast2GO software, and with the support of the refinement annotation strategies ANNEX and GO-Slim mapping for yeast and the InterProScan function (which detected the presence of domains in 4,187 sequences), 14,043 GO (gene ontology) terms were assigned to the 36,350 *P. pachyrhizi* unisequences. The transcripts were also submitted to the - Enzyme Code and KEGG function, which identified 1,519 contigs present in metabolic pathways, of which 1,129 demonstrated known enzymatic functions (Table 1).

### Gene ontology analysis and main functional categories

Blast2GO attributed a functional annotation to 15.47% of the *P. pachyrhizi* unisequences, resulting in 5,622 transcripts that were associated with GO terms. These sequences were classified and grouped according to their characteristics into three broad categories: cellular components, molecular functions and biological processes. A total of 1,934 cellular components, 4,010 molecular functions and 3,469 biological process terms were associated with our sequences (Table 2). The GO classifications were distributed in 15 levels among these three categories. The most informative GO level for *P. pachyrhizi* transcripts was level eight, which included a large number of annotated GO terms for biological processes.

**Table 2:** Summary of acyclic graphics for the arrangement of GO terms for cellular components, molecular functions and biological processes.

Categories	contigs	Categories	contigs	Categories	contigs
Cellular Component	1,934	Molecular Functions	4,010	Biological Processes	3,469
Membrane	697	Binding	2,289	Localization	609
part of membrane	474	Ion binding	968	transport	580
Cell	1,471	protein binding	574	single-organism localization	378
cytoplasm	573	organic cyclic compound binding	1,242	Biological Regulation	609
intracellular organelle	874	heterocyclic compound binding	1,242	regulation of biological process	383
ribonucleoproteins intracellular complex	338	small molecule binding	745	Single-Organism Process	1,693
Macromolecular Complex	854	carbohydrate derivative binding	557	single-organism localization	378
ribonucleoproteins complex	338	Catalytic Activity	2,353	single-organism cellular process	1,293
protein complex	530	transferase activity	596	single-organism metabolic process	1,150
Organelle	880	Hydrolase activity	745	Cellular Process	2,363
part of organelle	452	Oxidorredutase activity	474	regulation of the cellular process	365
Intracellular organelle	874			single-organism cellular process	1,293
non-membrane-bounded organelle	416			cellular metabolic process	1,848
membrane-bounded organelle	566			Metabolic Processes	2,693
				oxidation-reduction process	354
				cellular metabolic process	1,848
				primary metabolic process	1,853
				organic substances metabolic processes	2,041
				biosynthetic process	913
				nitrogen compound metabolic process	1,198
				Cell Component Organization or Biogenesis	437

Bold, underline, and simple font mean a progression from high to low hierarchical levels, respectively.

The cell component categories presented four parental terms: membrane (GO: 0.016.020), cell (GO: 0.005.623), macromolecular complex (GO: 0.032.991) and organelle (GO: 0.043.226) (Table 2). The parental term with the largest number of grouped sequences was cell, among which the sequences were related to parts of the cytoplasm and intracellular organelles, as well as components of the intracellular complex of ribonucleoproteins including ribosome sequences. The parental term organelles presented sequences related to intracellular organelles that were basically divided between organelles not delimited by membrane and organelles delimited by membrane; for the latter, there were sequences related to components of the nucleus. The parental terms membrane and macromolecular complex displayed limited detail for the sequences covered.

The molecular functions category presented only two parental terms: binding (GO: 0.005.488) and catalytic activity (GO: 0. 003 824) (Table 2). The parental term binding basically comprised sequences with molecular functions that were related to linkages between proteins, ionic bonds and linkage between nucleic acids, especially among purine bases. The parental term catalytic activity comprised sequences detailed only by exhibiting the metabolic activity of transferases, hydrolases, and oxidoreductases.

Finally, biological processes were the category that presented the largest number of classes of parental terms. A total of six parental terms were identified: location (GO: 0.051.179), biological regulation (GO: 0.065.007), single-organism process (GO: 0.044.699), cellular process (GO: 0.009.987), metabolic process (GO: 0.008.152), and organization of cellular component or biogenesis (GO: 0.071.840) (Table 2). Cellular process and metabolic process were the most strongly represented parental terms.

Cellular process presented the majority of sequences related to metabolic cellular processes including sequences that participate in heterocyclic metabolism, phosphorus metabolism, aromatic compounds, nitrogen compounds, cellular biosynthesis, and macromolecule metabolism (including protein metabolism and RNA metabolic processes). The majority of the sequences that were grouped into the parental term metabolic process were also related to metabolic cellular processes (including the sequences mentioned above) and to the metabolism of organic substances, which among others included the biosynthesis of organic compounds and macromolecules, as well as processes related to gene expression. The other parental terms within the biological processes were also related to the functions of some previously mentioned sequences, but they presented a lower level of detail of the identified sequences.

### Conserved domains and metabolic pathways

**Figure 1:**
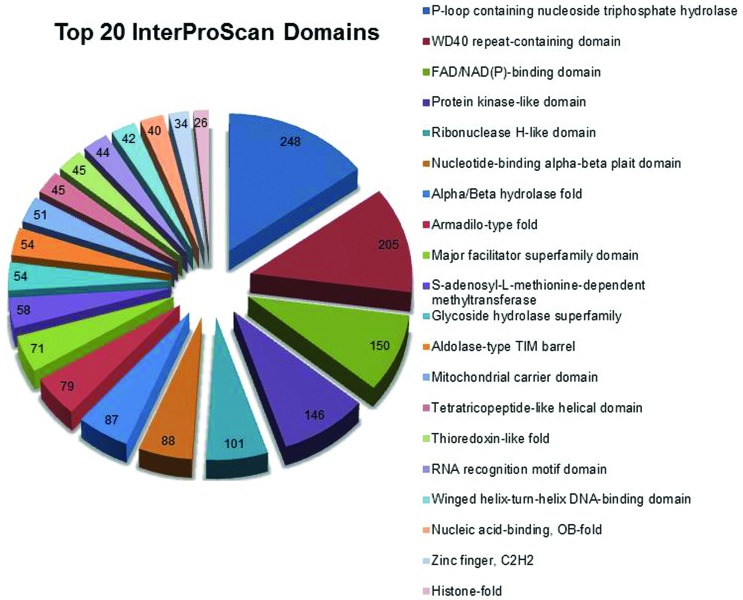
The most represented InterProScan domains associated with *P. pachyrhizi* transcripts. The number of sequences found for each domain is shown.

The InterProScan function in the Blast2GO software identified 4,187 non-redundant domains in the *P. pachyrhizi* unisequences (Figure 1). Among the 20 most abundant transcript domains identified, the main domain found among fungal transcripts was the P-loop domain containing nucleotide phosphate hydrolase (IPR027417), which was present in 248 sequences. Proteins that present this type of domain are usually responsible for catalyzing the hydrolysis of the phosphate binding present in the nucleoside triphosphate (NTP). The energy resulting from the hydrolysis of NTP is usually used to promote conformational changes in other molecules, forming the basis of the biological functions of most of these enzymes. The other identified domains cover different molecular functions such as oxidoreductase domains, protein-protein binding domains, nucleic acid recognition and cleavage domains, protein kinase domains, and transporter domains, among others.

Through the Enzyme Code and KEGG function available in the Blast2GO software, it was possible to identify metabolic pathways and enzymatic functions related to annotated transcripts of *P. pachyrhizi*, providing an overview of pathogen metabolism during interactions with the host. A total of 1,519 contigs were grouped into 99 metabolic pathways identified by KEGG. Some of these pathways were conspicuous because of the large number of transcripts distributed among different enzymatic classes, as observed for purine metabolism (ko00230), with 308 contigs and 26 enzymatic classes, and antibiotic biosynthesis (ko01130), with 96 contigs and 56 enzymatic classes. Among the other metabolic pathways, it was possible to identify different types of metabolism and processes, such as carbohydrate and lipid metabolism, cell cycle, cellular respiration processes, amino acid biosynthesis and degradation processes, protein interactions and regulation, biosynthesis of hormones, responses to stresses, and metabolic signaling pathway processes.

### The most highly expressed genes in the *P. pachyrhizi* transcriptome

Among the set of *P. pachyrhizi* 36,350 unisequences, the 50 most expressed transcripts, based on the FPKM values, were identified for each of the soybean genotypes (PI561356 and BRS 231) (Table S2). Among the 50 sequences identified for each genotype, 42 were common to both genotypes, resulting in the identification of 58 different transcripts that were not associated with any GO term. A total of 25 sequences presented annotations when aligned against the NR database and against the local database of phytopathogenic fungi, and these sequences showed similarities to the GAS1 and GAS2 virulence proteins of *M. grisea*. When 58 transcripts were compared to the secreted sequences identified by Link *et al.* (2014) in the haustorium transcriptome of *P. pachyrhizi*, it was possible to identify 44 sequences similar to 12 of the transcripts that were predicted as secreted. In addition, three transcripts that were found among the top expressed transcripts common to both genotypes (de_novo_2238, de_novo_5381, and de_novo_5849) were also functionally validated by Carvalho *et al.* (2016).

Some domains have been identified among the 58 most common transcripts present in almost all sequences, generally indicating their presence in membranes, as well as the formation of helices and coil structures (Coil – Coiled coil domain, Non-cytoplasmic domain, Transmembrane domain; Cytoplasmic domain, and TMhelix - Transmembrane helix domain). Eleven transcripts presented an uncharacterized domain related to a family of eukaryotic proteins named DUF3129 family (IPR021476). Another five domains were identified among 42 contigs common to both genotypes: the DNA polymerase III subunits gamma and tau (PRK07764); RNA polymerase I-associated factor PAF67 (PFAM10255); DUF70 domain (PFAM01901) present in archaebacteria and that may present transmembrane protein functions; MAEBL (PTZ00121) domain, which defines a family of erythrocyte-binding proteins present in malarial parasites that participate in host invasion; and finally a domain present in prokaryotic organisms related to membrane lipoproteins with specific lipid-binding properties (PS51257).

Specifically, for the most highly expressed *P. pachyrhizi* transcripts in contact with BRS231 (eight sequences), only three domains were found: a UL36 domain (PHA03247), which is typical of herpesvirus in tegument proteins, possibly acting shortly after the onset of infection; a fibronectin-attachment protein domain; and DNA polymerase III (PRK12323). Among the eight most highly expressed sequences detected only in PI561356, four domains were identified: a DUF3129 domain, as described above; a common fungal domain (IPR020100) encoding a protein with an as yet unknown function but related to the repression of carbohydrate metabolism, demonstrating increased levels during glucose breakdown; a non-catalytic Src homology (SH2) domain (PFAM14633) that is conserved among a series of cytoplasmic signaling proteins regulated by receptor protein-tyrosine kinases and is involved in normal signaling and cellular transformation; and an internal membrane protein (Tol-a) related to cell envelope integrity (PRK09510). Some non-specific domains have also been found among the 58 most expressed sequences, such as domains related to galactose-3-O-sulfotransferase proteins (Pfam06990), the synthesis of one form of natural vitamin k (cd13962), fibronectin binding proteins (Pfam07174), and keratin-associated proteins (Pfam11759), which are common domains in mammals.

### Enriched categories in the *P. pachyrhizi* transcriptome

We conducted an enrichment analysis in Blast2GO to identify GO terms that were overrepresented in each genotype for which *P. pachyrhizi* reads were obtained, susceptible (BRS231) and resistant (PI561356), as well as GO terms that were conserved and overrepresented among different infection structures, such as germinated urediniospores and appressoria (Stone *et al.*, 2012), haustoria (Link *et al.*, 2014), and leaf lesions.

**Figure 2:**
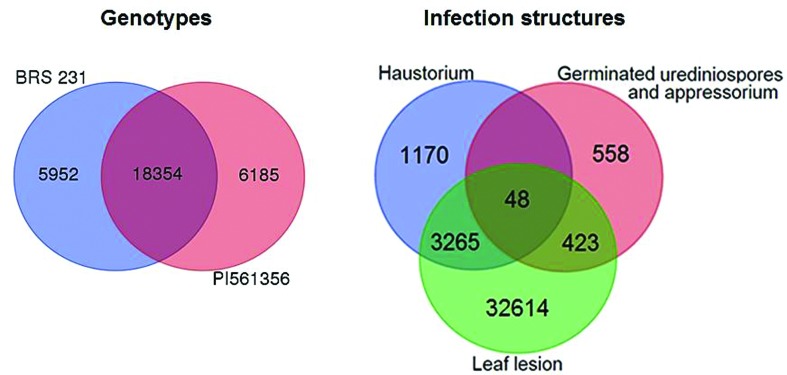
The number of *P. pachyrhizi* unisequences found exclusively among susceptible and resistant genotypes (BRS231 and PI561356, respectively) and common among different fungal infection structures (germinated urediniospores and appressorium, haustorium, and leaf lesion).

Most of the 36,350 *P. pachyrhizi* unisequences were generated by assembling reads obtained from the two genotypes; however, some contigs were exclusive to reads obtained from susceptible (BRS231) or resistant (PI561356) genotypes. Considering only transcripts with FPKM values equal to or greater than one (30,491 contigs), it was possible to identify 5,952 contigs that were composed exclusively of reads from *P. pachyrhizi* genes expressed in BRS231, and 6,185 contigs composed exclusively of reads from *P. pachyrhizi* genes expressed in PI561356 (Figure 2). Among the 5,952 sequences found exclusively in the susceptible genotype, 1,349 presented hits in the GenBank non-redundant (NR) database and 456 were associated with GO terms. Of the 6,185 sequences found exclusively in the resistant genotype, 1,904 presented hits in the GenBank NR database, and 243 were associated with GO terms. Between both genotypes, most contigs were similar to hypothetical or predicted proteins of *Puccinia* and/or *Melampsora*.

To verify the presence of transcripts common to different infection structures, 1,029 sequences expressed in germinated urediniospores and appressoria (Stone *et al.*, 2012) and 4,483 sequences expressed in haustoria (Link *et al.*, 2014) were aligned against the *P. pachyrhizi* unisequences generated in this work, which corresponded to sequences expressed in leaf lesions. A total of 3,265 sequences from the dataset of haustoria and 423 sequences from the dataset of germinated urediniospores and appressoria showed a high similarity to *P. pachyrhizi* unisequences; of these two datasets, 48 were common to all the structures (Figure 2). Of the transcripts that were common to haustorium and leaf lesion sequences, only 154 of the 3,265 sequences were associated with GO terms. For the transcripts common to germinated urediniospore, appressorium and leaf lesion sequences, 12 of the 423 sequences were associated with GO terms. Finally, of the 48 transcripts that were common to all the infections structures, eight sequences were associated with GO terms.

### Enriched categories among the genotypes

The enrichment analyses performed with the transcripts from each genotype against the total set of *P. pachyrhizi* unisequences (36,350 contigs) revealed no enrichment of molecular classes among the sequences found in BRS231, whereas for PI561356, it showed 23 different enriched molecular classes. Although no enriched classes were found between BRS231 and the total transcriptome, it was possible to observe sequences that were exclusive to the susceptible genotype, which comprised four different and general molecular classes for 456 transcripts associated with GO terms: catalytic activity, protein binding, metal cluster binding, and metabolic process.

**Figure 3:**
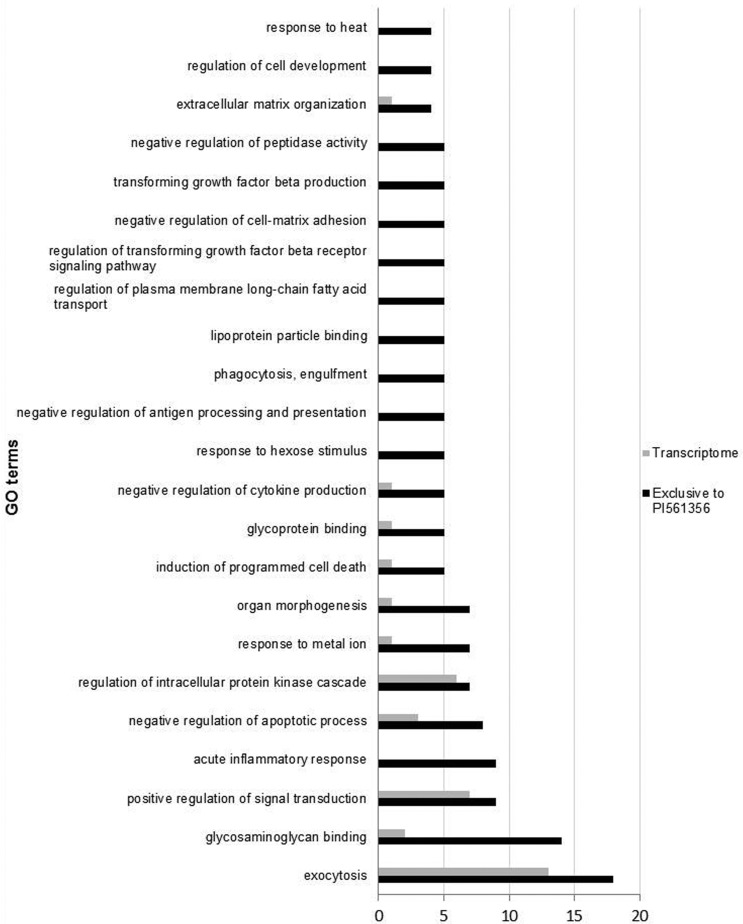
Differential gene ontology (GO) term distribution among the *P. pachyrhizi* transcriptome (36,350 contigs) and transcripts generated exclusively in the resistant genotype PI561356 (6,185 contigs). This figure presents only the molecular classes enriched for the exclusive transcripts of PI561356. The graph was generated automatically after the enrichment analysis using the BLAST2GO tool (Fisher’s exact test, *p* < 0.05).

Among the 23 enriched molecular classes among the transcripts from the resistant *P. pachyrhizi* genotype, the majority were related to fungal pathogenic activity during host infection. The class related to exocytosis activity had the largest number of contigs (18 sequences), followed by glycosaminoglycan binding (14 sequences), positive regulation of signal transduction (nine sequences), and acute inflammatory response (nine sequences). Other molecular classes also stood out from the others; for example, regulation of the intracellular protein kinase cascade and extracellular matrix organization. The enriched molecular classes, as well as the number of sequences present in the resistant genotype (PI561356) from the identified GO terms, are shown in Figure 3.

The other 16 molecular classes identified among the transcripts, but not enriched for sequences from the resistant genotype, comprised different biological activities related to, for example, the ribonucleoprotein complex, which contained the largest number of contigs (82 sequences), in addition to integral membrane components, transcription processes, nucleic acid biosynthesis, RNA processing, phosphorylation and protein transport, and regulation of cellular component organization, among others.

### Enriched categories among fungal structures

**Figure 4:**
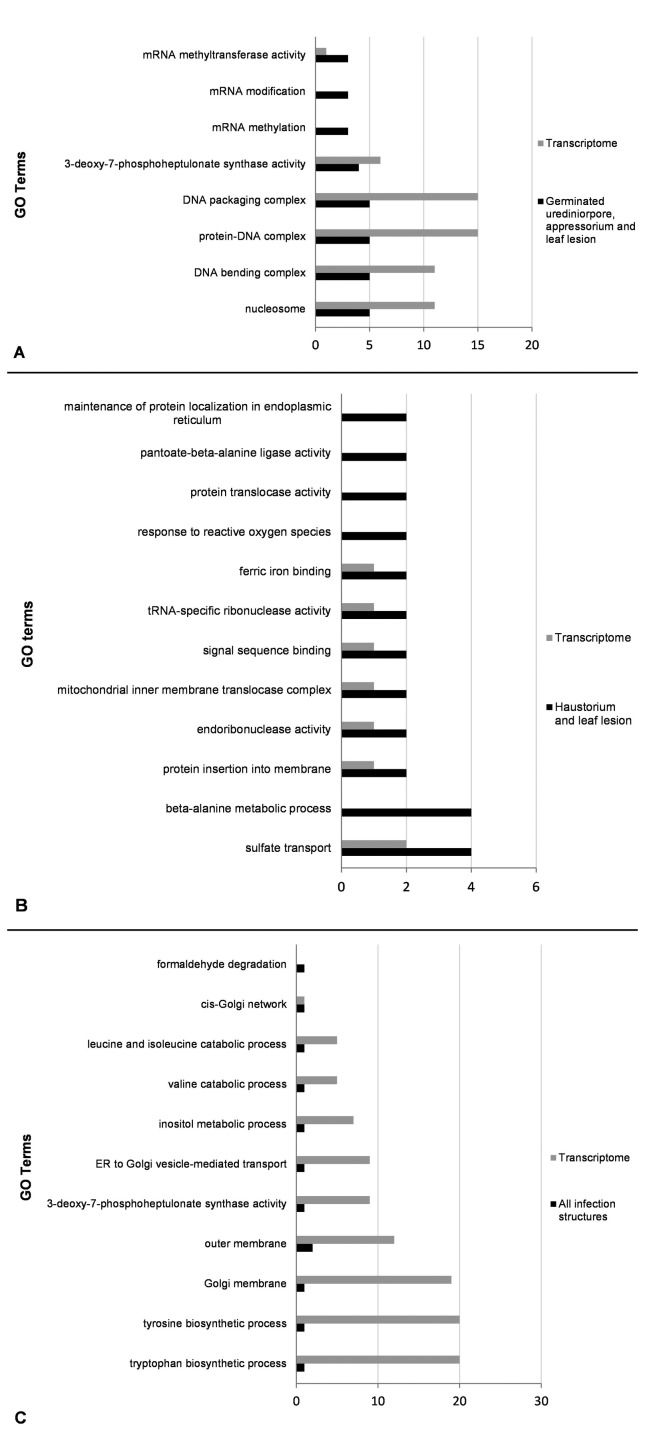
Differential gene ontology (GO) term distribution between the *P. pachyrhizi* transcriptome (36,350 contigs) and fungal infection structures. (A) Transcripts common to germinated urediniospores, appressoria, and leaf lesions (423 contigs); (B) transcripts common to haustoria and leaf lesions (3,265 contigs); (C) transcripts common to all infection structures, germinated urediniospores, appressoria, haustoria, and leaf lesions (48 contigs). Graph B shows only the molecular classes enriched for transcripts common to haustoria and leaf lesions. The graph was automatically generated after the enrichment analysis using the BLAST2GO tool (Fisher’s exact test, FDR adjusted *p*-value < 0.05).

The enrichment analyses performed with transcripts common to different infection structures against the *P. pachyrhizi* transcriptome (36,350 contigs) allowed the identification of three enriched molecular classes among sequences common to germinated urediniospores, appressoria and leaf lesions, 12 enriched molecular classes between sequences common to haustoria and leaf lesions, and no enriched molecular class was observed between sequences common to all the analyzed infection structures (Figure 4).

The 12 transcripts that were common to germinated urediniospores, appressoria and leaf lesions, which were associated with GO terms, were classified into eight different molecular classes comprising the modification and methylation of mRNA molecules, DNA binding and packaging complexes, protein-DNA complexes, nucleosomes, and enzymes that function in the shikimate pathway. For this analysis, only classes associated with mRNA modifications (modification and methylation of mRNAs and mRNA methyltransferase activity) were enriched in relation to the *P. pachyrhizi* transcriptome, with less than five sequences in each class (Figure 4A).

Of the 154 transcripts associated with GO terms common to haustoria and leaf lesions, a total of 34 molecular classes were identified. Among the 12 enriched classes, the largest number of sequences was related to sulfate transport and beta-alanine metabolic processes, each of which contained four contigs. In addition to these classes, it was also possible to identify molecular classes related to the response to reactive oxygen species, endoribonuclease activity, and protein translocase activity, among others. The remaining 22 molecular classes included, for example, molecular functions related to different biosynthetic processes, oxidation-reduction processes, ribonucleoprotein complexes, intracellular transport, the generation of precursor metabolites and energy, transcription processes and the modification of RNA (Figure 4B).

Finally, eight of 48 transcripts that were common to all infection structures were associated with GO terms and classified into 11 molecular classes, but none was significantly (FDR *p*-value < 0.05) enriched in relation to the transcriptome. The molecular classes identified encompassed sequences that were basically related to catabolism and the biosynthesis of amino acids, as well as the transport of substances between the endoplasmic reticulum and the Golgi complex (Figure 4C).

Among the 40 remaining transcripts that were common to all infection structures and that were not associated with GO terms, 15 were not annotated against the NCBI database. Among the transcripts that were annotated in the NCBI database, 14 sequences were similar to *M. larici-populina* and/or *P. graminis* hypothetical proteins, two sequences were similar to *P. pachyrhizi* clones, one sequence was related to a secreted protein from *M. larici-populina*, and one sequence was similar to the carbohydrate esterase family of *M. larici-populina*. Seven transcripts presented non-specific annotations.

### Comparative analysis

Predicted protein sequences from 13 fungal genomes and two oomycetes genomes were grouped with the *P. pachyrhizi* unisequences (36,350 transcripts) based on the similarity of the sequences, thus constituting OrthoMCL gene families. We identified 4,775 OrthoMCL multigenic families containing at least two sequences. The OrthoMCL families comprised 52,380 protein sequences, of which 6,072 were *P. pachyrhizi* transcripts, accounting for 16.7% of the total fungal transcripts.

The correlations between sequences of *P. pachyrhizi* and the other 15 species used for the construction of OrthoMCL families were analyzed, and the highest values were observed for the families that were common to all species (with 260 OrthoMCL families and 5,475 genes distributed among them) and families common to rust-causing fungi (with 92 OrthoMCL families and 525 genes distributed among them), followed by families that were common to basidiomycetes (with 7 OrthoMCL families and 130 genes distributed among them) (Table 3).

**Table 3:** General characteristics of the comparative analysis between the OrthoMCL multigene families obtained from the predicted proteins of the *P. pachyrhizi* transcriptome and proteins predicted from other 15 species.

Molecular categories of OrthoMCL families	OrthoMCL families	Total of sequences	*P. pachyrhizi* sequences
*Families common to all species* ^1^			
*Ribosomal proteins*	54	1,014	75
*Predicted proteins*	24	449	28
*Protein synthesis*	17	309	18
*Dehydrogenases*	11	311	17
*Cytoplasmic transporters*	8	225	12
*Hypothetical proteins*	5	87	8
*Membrane transporters*	2	334	7
*Others*	139	2,746	169
Total	260	5,475	334
*Families common to basidiomycetes*			
*Predicted proteins*	2	32	3
*Hypothetical proteins*	1	22	1
*Secreted proteins*	1	35	7
*Transcription factor binding domains*	1	17	1
*Methylation*	1	12	1
*Metabolic pathways signaling*	1	12	1
Total	7	130	14
*Families common to rust fungi*			
*Hypothetical proteins*	66	374	77
*Secreted proteins*	11	93	17
*Carbohydrate metabolism*	4	21	4
*Predicted proteins*	3	14	4
*Transport of peptides*	1	10	1
*Spindle checkpoint signaling*	1	6	1
*Protein metabolism*	1	5	1
*Signaling and regulation of the circadian cycle*	1	5	1
*Vesicular fusion*	1	4	1
*Transmembrane transport*	1	4	1
*Nitrogen metabolism*	1	4	1
*No annotation*	1	5	1
Total	92	525	110
*Families* *exclusive of P. pachyrhizi* ^2^			
*No annotation*	510	867	867
*Hypothetical proteins*	101	179	179
*Predicted proteins*	45	80	80
*Secreted proteins*	8	18	18
*Others*	192	316	316
Total	856	1,460	1,460

1 Families common to all species: for this parameter only the eight molecular categories were listed that had the largest number of families, or a greater number of sequences.

2 Families exclusive of *P. pachyrhizi*: for this parameter only the four molecular categories were listed that had the largest number of families, or a greater number of sequences.

Hypothetical proteins and predicted proteins were the molecular categories corresponding to the largest portion of observed sequences, excluding families common to all species that presented ribosomal proteins as the most abundant sequences. Secreted proteins were observed for families common to basidiomycetes and families common to rust fungi. For both parameters, the number of secreted sequences corresponded to approximately 25% of all sequences found in the families. Specifically, for the families of secreted proteins among the 12 species of basidiomycetes, *P. pachyrhizi* was the species with the greatest number of sequences, presenting 7 of the 14 predicted secreted sequences. The other families were mainly characterized by involvement in primary metabolic processes related to the survival and maintenance of the organism, such as protein and carbohydrate metabolism, the transport of substances, and a few families involved in secondary metabolism processes such as metabolic signaling pathways.

The molecular category with the largest number of families, representing 18.2% of the sequences in the families common to all species, was that of genes encoding ribosomal proteins (mainly for the 60S and 40S subunits), with 75 sequences identified in *P. pachyrhizi*. Other categories also presented a large number of sequences, such as proteins involved in the translation process (mainly translation factors and tRNAs), in addition to general dehydrogenases and transporters. Two families specific for ABC membrane transporters predominated as the two largest families common to all species in relation to the number of sequences, comprising 334 genes. The 139 families common to all species that are not presented in the table include genes involved in the most diverse biological processes, including the metabolism of carbohydrates, proteins, nucleic acids and energy metabolism, transcription processes, and metabolic signaling pathways, among others.

Few families were common among the species of basidiomycetes, but the identified families were related to interesting functions such as transcription processes, nucleic acid methylation processes, and signaling pathways. Rust fungi shared families with different molecular functions, most of which were related to general processes of maintenance and cell survival, such as protein and carbohydrate metabolism, spindle checkpoint signaling, and peptide transport. Other families may have a more direct relationship with pathogenicity processes that are common to these species, such as families related to transmembrane transporters and vesicle fusion.

Among the sequences found in *P. pachyrhizi*, some were similar to those found in the haustorium transcriptome results obtained by Link *et al.* (2014). Most were observed in families that were common to all species, constituting a total of 97 sequences performing various functions, from sequences related to histone proteins and cell cycle proteins to proteins related to metabolic signaling pathways. In the families common to rust fungi, 20 sequences were found, of which 14 were in the hypothetical protein category, four were identified as secreted proteins, and two others were related to protein metabolism and circadian cycle signaling and regulation.

Families that were exclusive to *P. pachyrhizi* were also identified, making up a total of 856 families encompassing 1,460 genes, of which 422 sequences were identified among the transcripts obtained by Link *et al.* (2014). Among the families exclusive to *P. pachyrhizi*, most did have functional annotations (510 families); however, among those with a known function, the presence of gene families could be observed beyond those identified as hypothetical, predicted, or secreted sequences. Of the 18 sequences found in families of secreted proteins, two (de_novo_3939 and de_novo_7164) belonging to the same family were also present in the results of the functional validation reported by Carvalho *et al.* (2016). The other gene families that are not shown in the table presented the most varied functions, among which we have highlighted sequences that function in carbohydrate metabolism, protein and nucleic acid metabolism, cell cycle, and splicing processes, such as translation factors, metabolic signaling pathways, and membrane transporters (including MFS and ABC transporters).

### Prediction of transcriptionally active transposable elements

BLAST analyses were performed against a reference database of transposable elements (Repbase) using the computer tool RepeatMasker and resulted in the identification of 592 contigs with transposable elements (TE) fragments. Among these contigs, 484 were annotated as retrotransposons (Class I, 81.76%) and 108 as DNA transposons (Class II, 18.24%) (Table 4). Among class I, 413 elements were classified as long terminal repeat elements (LTR elements), and 132 elements were classified as non-long terminal repeat elements (non-LTR elements) and, more specifically, as long interspersed nuclear elements (LINEs). Among class II, 90 elements were classified as terminal inverted repeats elements (TIR elements), two as Cryptons, and 22 as Helitrons.

**Table 4:** Transcriptionally active transposable elements in *P. pachyrhizi* transcriptome.

TE classification	Nr. of TE elements	Nr. of *P. pachyrhizi* contigs
Class I (retrotransposons)	545	484
*LINE*	*132*	*98*
Tad1	129	96
Deceiver	2	2
L1	1	1
*LTR*	*413*	*386*
Copia	214	195
Gypsy	188	183
Class II (DNA transposons)	124	108
*TIR*	*90*	*85*
Tc1-Mariner	37	35
PIF-Harbinger	26	24
EnSpm	18	18
Zisupton	3	3
P-Fungi	1	1
Merlin	2	2
hAT-Ac	2	2
MuDR (MULE)	1	1
*Helitron*	*22*	*20*
*Crypton*	*2*	*2*

Underline, italic, and simple fonts represent the classes, orders and superfamilies, respectively.

Different superfamilies of TEs were found within the orders of each class. In class I, the LINE elements presented three different superfamilies, the Tad1, Deceiver, and L1 superfamilies, and the LTR elements consisted of the Copia and Gypsy superfamilies. The Copia and Gypsy superfamilies were the most abundant mobile elements in the fungal genomes. In class II, the TIR elements presented eight different superfamilies: Tc1-Mariner, PIF-Harbinger, EnSpm, Zisupton, P-Fungi, Merlin, hAT-Ac, and MuDR (MULE). In addition to the transposable elements found among the unisequences of *P. pachyrhizi*, 10,012 simple repeat elements were also identified and another detected 1,376 elements that were not identified because they were classified as low complexity sequences.

### Validation of RNA-Seq expression levels by RT-qPCR

RT-qPCR was performed using six *P. pachyrhizi* contigs that were preferentially selected because of their involvement in pathogenicity, showing expression levels at different time points of *P. pachyrhizi* infection that were consistent with the expression levels revealed by the FPKM values from the mRNA-Seq data (Table 5). The expression levels identified by RT-qPCR for the Thi (thiamine biosynthesis), PPI (peptidyl-prolyl-cis/trans isomerase), AGO (argonaut), Pv-SNARE (soluble NSF attachment receptor), and HSS (small heat shock) genes revealed that these genes were induced at some point during the process of plant infection by the pathogen. Similar results were determined for the transcripts of these genes by RNA-Seq, which showed high normalized FPKM values.

**Table 5:** Validation of gene expression base on mRNA-Seq assay using RT-qPCR.

Genes	mRNA-Seq^3^		RT-qPCR^1^,^2^									
	BRS231	PI561356	ES	EG	0hpi	6hpi	12hpi	24hpi	48hpi	72hpi	96hpi	192hpi
Thi	27.146	23.533	0.004	0.011	0.187	0.003	0.052	0.467	1.054	0.843	0.894	1.589
PPI	21.178	24.666	0.212	0.412	0.282	0.082	0.392	1.118	1.211	1.386	1.658	2.340
AGO	5.676	6.886	1.083	2.075	0.854	1.613	3.075	2.773	1.157	1.048	1.375	2.782
Pv-SNARE	3.209	5.561	2.636	8.464	3.144	7.926	5.174	0.829	0.397	0.293	0.268	0.191
HSS	6.780	3.387	0.144	0.457	1.245	1.138	2.985	1.103	1.677	1.617	1.204	0.836
NtR	0.288	0.356	0.097	0.200	0.117	0.140	0.389	0.110	0.272	0.191	0.195	0.231

1 Main *P. pachyrhizi* infection time points: at the stages of spore (ES) and germinated spore (EG) before contact with soybean, and after soybean contact at 0, 6, 12, 24, 48, 72 , 96 and 192 hours post infection “hpi”.

2 qPCR results are represented by fold change values obtain after normalization with the endogenous tubulin gene.

3 mRNA-Seq results are represented by FPKM values obtained after normalization with the endogenous tubulin gene.

The highest expression levels based on the mRNA-Seq results for induced genes were observed for the Thi and PPI genes, which demonstrated standardized FPKM values greater than 20, and the lowest values were observed for the Pv-SNARE gene with FPKM values less than 6. In contrast, the highest RT-qPCR values were obtained for the Pv-SNARE gene, with fold change values up to 8.4, and the lowest values were observed for the Thi gene, with fold change values up to 1.6. Specifically for the NtR (nitrate reductase) gene, both the expression levels by mRNA-Seq and by RT-qPCR showed that, unlike other contigs, this one presented low expression levels. These results showed that the RT-qPCR results at different time-points did not exactly align with the mRNA-Seq values; however, despite these differences in expression levels, the RT-qPCR expression values for all genes tested were consistent with those provided by the mRNA-Seq data.

## Discussion

By using the combination of the LCM technique, high-throughput sequencing, and the merge of *P. pachyrhizi* NCBI ESTs with our contigs, we generated 36,360 *P. pachyrhizi* unisequences. The total number of transcripts obtained corresponded to 73.3% of the total *P. pachyrhizi* sequences available in NCBI (49,596 ESTs), but only 23,290 contigs showed similarity between these sequences, suggesting that approximately 36% of the transcripts obtained in this work may still be unknown. Although a high level of similarity with previously identified transcripts was observed, it is noteworthy that most of these transcripts lacked a functional annotation. Additionally, the results suggested that approximately one-third of the generated sequences could be conserved since they presented similarities to proteins encoded by other phytopathogenic fungi. These similarities were more specific among other rust fungi such as *P. graminis* and *M. larici-populina*, for which more than half (56% and 57%, respectively) of their predicted sequences (Duplessis *et al.*, 2011) were similar to the transcripts of *P. pachyrhizi* obtained herein.

Functional annotations were assigned to a total of 5,622 contigs corresponding to only 15.5% of the identified transcripts, but this number of sequences was similar to that observed for other rust species (Garnica *et al.*, 2013) and three times greater than that obtained by Link *et al.* (2014) for the *P. pachyrhizi* haustorium transcriptome. In addition, although the total number of *P. pachyrhizi* transcripts identified herein was large (36,360 unisequences), the number of genes encoding proteins is expected to be much less, similar to that identified for other rust species such as *M. larici-populina* and *P. graminis* with 16,399 and 17,773 predicted protein-coding genes in their genomes (Duplessis *et al.*, 2011). However, the lack of access to the whole genome of *P. pachyrhizi* impairs an understanding of its composition and functions.

Most of the molecular processes and metabolic pathways observed in our study have already been described for other fungi, including rust fungi and *P. pachyrhizi* during the main stages of the infection process. Energy and carbohydrate metabolism were also reported by Tremblay *et al.* (2013), who detected genes encoding enzymes involved in these metabolic processes from non-germinated urediniospores until the moment of sporulation. In the same study, after contact of the non-germinated urediniospores with the host until the beginning of the germination process, the presence of transcripts involved in oxidative phosphorylation processes and transcription processes suggested a high level of energy production dedicated to the transcription of genes involved in subsequent stages required for pathogen development.

Transcriptome analysis of *P. striiformis* (Garnica *et al.*, 2013) and *P. pachyrhizi* (Posada-Buitrago and Frederick, 2005; Tremblay *et al.*, 2013; Link *et al.*, 2014) demonstrated that nucleic acid metabolism (mainly DNA synthesis), transcription processes, cell cycle control, and metabolic signaling pathway processes function during the germination of urediniospores. In addition, it is believed that *P. pachyrhizi* does not have access to host nutrients in the early stages of infection; therefore, glycerol (necessary for penetration) is synthesized from the nutrients obtained from lipids, glycogen, and sugar catabolism present in urediniospores, indicating the activity of these metabolic pathways in the degradation of these compounds during germination until penetration of the host tissue (Thomas *et al.*, 2002; Both *et al.*, 2005).

Transcripts related to processes ranging from the uptake of sugars and amino acids (membrane transporters and carbohydrate metabolism), as well as lipid metabolism to more active biosynthetic and transcription processes are mainly observed during haustorium formation (Both *et al.*, 2005; Tremblay *et al.*, 2013; Link *et al.*, 2014). However, carbohydrate and lipid synthesis, as well as protein synthesis and amino acid metabolism, are also involved in the later stages of fungus development during uredinia formation and later during sporulation (Both *et al.*, 2005; Tremblay *et al.*, 2013). Nucleic acid metabolism in *P. pachyrhizi* is mainly represented by transcripts associated with RNA metabolism and DNA replication and repair. Both processes reflect the proliferation of the fungus through the synthesis of proteins and cell division, which are mainly involved in the process of penetration and the production of urediniospores, as observed for the proteome of hyphae during the sporulation of *B. graminis* (Bindschedler *et al.*, 2009).

The metabolism of purines, which was found to make up the largest number of transcripts identified by KEG analysis, was described in *M. oryzae* as essential for the growth of the fungus in the host cell (Fernandez *et al.*, 2013). In this study, a mutant for the SAICAR synthetase-encoding gene (*MoADE1*) showed no differences in appressorium formation or rice cuticle penetration compared to wild type, but it exhibited a larked reduction in pathogenicity on rice leaves compared with wild type, indicating that *de novo* adenine biosynthesis is essential for disease development by *M. oryzae.* The authors suggest that the attenuated pathogen growth in rice cells observed for mutant strains may be due to an impaired ability of the fungus to obtain more complex molecules such as purines, unlike sugars, from host cells via the invaginated plant-derived plasma membrane, called the extra-invasive hyphal membrane (EIHM).

Transcripts related to nitrogen metabolism potentially function after haustorium formation during the assimilation of compounds from the host. Genome and transcriptome analysis of *M. lini* after the development of haustorium, at six days after inoculation, revealed the presence of a putative gene for nitrate reductase. However, no transcripts were found to possess this function, indicating that this metabolic pathway may not be functional in this species (Nemri *et al.*, 2014), as predicted for most species of rust (Spanu *et al.*, 2010, Cantu *et al.*, 2011). However, in the same study, Nemri *et al.* (2014) identified homologues of the ammonia assimilation pathway, suggesting that most of the nitrogen acquired from the host is assimilated in the form of ammonia. These results corroborate the findings of the present work, in which the sequence related to the nitrate reductase gene showed low expression levels in both RNA-Seq and RT-qPCR analyses, and the nitrogen metabolism transcripts identified by KEGG functioned at the end of the assimilation pathway for this compound, and more specifically, for ammonia compounds (data not shown). In addition, the OrthoMCL family analyses revealed that some genes related to nitrogen metabolism were conserved among the rust species used in this work.

Among the 58 most highly expressed transcripts in the transcriptome analysis, almost half showed similarity to the virulence proteins GAS1 and GAS2 of *M. grisea*. These proteins are virulence factors that function mainly in the initial stages of the infection process during the penetration of host tissues and have been previously identified in other fungi including the *P. pachyrhizi* secretome (Xue *et al.*, 2002; Carvalho *et al.*, 2016). The DUF3129 domain was one of the most abundant domains found among the most highly expressed transcripts, and although its function is still unknown, it appears to be conserved among some species of rust and has also been identified more specifically among sequences secreted by these fungi, as observed for *P. graminis* and *M. larici-populina* (Saunders *et al.*, 2012) and even for *P. pachyrhizi* (Stone *et al.*, 2012, Carvalho *et al.*, 2016). The SH2 domain found among the most highly expressed transcripts for the resistant genotype (PI561356) is a type of phosphotyrosine signaling factor and, despite being rarely found in fungi, it has been described to play a central role in many cell-to-cell communication pathways, including those that regulate proliferation, differentiation, adhesion, hormone responses, and immune defense (Hunter, 2009; Lim and Pawson, 2010). Additionally, three of the most highly expressed transcripts common to both genotypes (de_novo_2238, de_novo_5381, and de_novo_5849) were also analyzed by Carvalho *et al.* (2016) as putative *P. pachyrhizi* effectors and were functionally validated by transient overexpression in tobacco leaves, revealing the ability of these sequences to suppress ETI responses.

For the enrichment analyses, the transcripts expressed in the resistant genotype PI561356 presented enriched molecular categories that were closely related to the process of host tissue infection. In addition to the basic mechanisms underlying the development of the pathogen such as the regulation of cellular development, organization of the extracellular matrix, and regulation of growth factor pathways, transcripts related more closely to the pathogenicity of the fungus, such as processes of cellular secretion and metabolic signaling pathways, were also enriched. The process of exocytosis or cellular secretion is essential during infection of host tissues, among other reasons, mainly with respect to the secretion of proteins called effectors during haustorium formation (Catanzariti *et al.*, 2010). These effector proteins are responsible for altering the structure and function of the host cell, leading to molecular and physiological changes that facilitate infection and nutrient uptake by the pathogen (Voegele and Mendgen, 2011). Several putative effector proteins have been identified in oomycetes, fungi, and rust fungi, some of which have previously been shown to be directly related to the infection process, such as in *B. graminis* (Pliego *et al.*, 2013), in *P. infestans* (Sanju *et al.*, 2015) and even in *P. pachyrhizi* (Carvalho *et al.*, 2016).

In addition to the processes of cellular secretion, the molecular categories of positive regulation of signal transduction and regulation of intracellular protein kinase cascades that were enriched in PI561356 may be associated with a metabolic signaling pathway that is induced in response to defense mechanisms acting on the host cell. Mitogen-activated protein kinases (MAPKs) are one of the most well-known types of kinases. MAPKs generally influence the transmission of stress signals from receptors to specific effectors that regulate gene expression, cell growth, and differentiation during the various processes underlying the development and adaptation of different organisms (Moustafa *et al.*, 2014). Silencing of a gene encoding a MAPK in *P. triticina* led to disease suppression in the host, revealing the intimate relationship of this gene with the pathogenicity of this rust fungus (Panwar *et al.*, 2013).

Different molecular classes were also enriched among transcripts common to *P. pachyrhizi* infection structures. Modification and methylation of mRNA molecules were enriched processes among the sequences found in germinated urediniospores, appressoria, and in transcripts obtained from leaf lesions. These molecular classes indicate that the common transcripts among these structures are basically involved in transcription and mRNA processing processes. As previously mentioned, transcription processes are very active during most of the infection process, mainly during the stages from germination up to penetration of the host tissue.

When we compared our transcriptome to *P. pachyrhizi* germinated urediniospores and appressorium ESTs and to haustorium transcripts, it was possible to access transcripts related to all these biological structures, with a predominance of haustorium-related transcripts. This finding was expected because, in addition to the greater number of sequences derived from the haustorium transcriptome dataset (Link *et al.*, 2014), the isolation of mesophyll cells from immediately below the rust lesions probably resulted in an enrichment of haustorium structures at 10 days after inoculation. At this time, the pathogen had already completed one reproduction cycle and started the next one. Hacquard *et al.* (2010), also using the LCM technique, isolated different portions of uredinia formed by *M. larici-populina* on susceptible poplar leaves, such as spores and sporogenous hyphae, as well as fungus-infected spongy mesophyll and palisade mesophyll tissues. The exon oligo arrays were used to measure the transcript expression in these areas, and the results revealed gene expression associated with biotrophy in the last tissue. Among these sequences, a massive induction of sequences encoding putative effector proteins were identified, supporting the maintenance of biotrophy during late infection stages. As reported by these authors, the use of LCM to collect samples provided good preservation of plant and fungal cell structures, thus maintaining the integrity of RNA isolated from microdissected tissues.

Among the categories enriched for common transcripts between haustorium sequences and leaf lesions, the molecular categories of sulfate transport predominate. Sulfate is usually taken up by fungi and then converted to a precursor of cysteine (Marzluf, 1997), but some rust fungal species such as *B. graminis* (Spanu *et al.*, 2010) and *P. graminis* (Duplessis *et al.*, 2011) lacked genes encoding enzymes related to sulfate uptake and reduction in their genomes. Regardless, Garnica *et al.* (2013) and Castillejo *et al.* (2010) found evidence of sulfur metabolism in *P. striiformis* and *Uromyces striatus*, respectively, corroborating our results.

Another interesting enriched molecular category is the response to reactive oxygen species (ROS). In plants, ROS are known to modulate defense mechanisms against pathogen infection, including programmed cell death (Dangl and Jones, 2001). ROS are generated by several different enzymes, with NADPH oxidase being one of the most well-known, and in fungi, ROS are involved in the regulation of a variety of cellular physiological and differentiation processes such as defense and infection processes (Takemoto *et al.*, 2007). Functional analyses following deletions of the single NADPH oxidase gene from *Podospora anserina* (*Nox1*) (Malagnac *et al.*, 2004) and *Neurospora crassa* (*nox-1*) (Aguirre *et al.*, 2005) demonstrated that the production of ROS is critical for sexual fruiting body development in filamentous fungi. In *M. grisea*, ROS production was observed mainly during appressorium development, and two genes encoding NADPH oxidases were found to be required for the pathogenicity of this fungus (Egan *et al.*, 2007). Sequences similar to NADPH oxidases have previously been identified among *P. pachyrhizi* transcripts obtained from germinated urediniospores and appressoria (Stone *et al.*, 2012).

A comparative analysis between *P. pachyrhizi* and 15 other species of fungi and oomycetes revealed that the largest number of conserved sequences among these species was grouped into ribosomal protein families. Ribosomal proteins play an important role in all organisms allowing translation, and among the large number of families, many are conserved among species of Bacteria, Archaea and Eucarya (including fungi species) (Lecompte *et al.*, 2002). Among eukaryotic organisms, a phylogenetic analysis involving sequences of ribosomal proteins showed that families of these proteins present in Plantae and Animalia species are more closely related than other detected fungal species, which form a more distant clade (Veuthey and Bittar, 1998). In addition, Tanay *et al.* (2005) suggest that, specifically in fungi, some coregulated responses related to ribosomal proteins may be conserved even though the underlying regulatory mechanisms are changing, which can be explained by the formation of a redundant intermediate program. These results allow us to infer that in addition to conserving the sequences and structures of ribosomal proteins, other mechanisms may be involved in maintaining the function of these proteins among different fungal species.

The two families of membrane transporters identified among all species also deserve attention in this discussion because of the high sequence numbers they contained. Both were families of ABC (ATP binding cassette) membrane transporters, which play an important role in the transport of various substances (Rees *et al.*, 2009) and have been described previously in different fungal species. In *P. striiformis,* twelve transcripts similar to ABC transporters were identified, and although it has not been possible to establish their biological functions, three of these sequences were upregulated in germinated spores (Garnica *et al.*, 2013). In *M. oryzae*, three ABC transporter genes (*ABC1*, *ABC3* and *ABC4*) seem to be directly related to mechanisms of fungal pathogenicity during appressorium formation and penetration of the host tissue, reflecting the possible role of these sequences in the protection of pathogen cells, excluding defense molecules secreted by plants, as well as the secretion of secondary metabolites, which are important for colonization of the host tissue (Urban *et al.*, 1999; Sun *et al.*, 2006; Gupta and Chattoo, 2008; Soanes *et al.*, 2012).

For the rust fungi used in the comparative analysis, conserved sequences among species were identified in multigene families related to carbohydrate and protein metabolism, transmembrane transport, and vesicular fusion. Sequences related to the first three molecular processes have been previously identified in other phytopathogenic fungi, including rust fungi, during development of the main infection structures. The sequences involved in the vesicular fusion process are required for the development of *U. maydis* and *M. oryzae* in host tissue for their pathogenicity, impacting both uptake and secretion mechanisms (Fuchs *et al.*, 2006; Qi *et al.*, 2016). Carvalho *et al.* (2016) further demonstrated that two sequences of putative effector proteins present in the same secreted protein family, which was conserved among fungi in our analysis, are able to suppress ETI responses in tobacco leaves by overexpression.

Transposable elements can drastically interfere with the composition and expression of a genome. The movement of transposons or retrotransposons into or near genes can contribute to partial or total gene inactivation, impacting the regulation of gene expression and potentially still contributing to a large genotypic and phenotypic variety. In recent decades, knowledge about TEs in fungi has increased greatly due to the growing number of studies involving fungi of medicinal, agronomic, and biotechnological importance, including filamentous fungi (Daboussi and Capy, 2003). TEs were identified in sequenced genomes of other rust fungi such as *B. graminis*, in which the TEs correspond to 64% of the genome size (Spanu *et al.*, 2010), *M. larici-populina* and *P. graminis*, in which the TEs account for approximately 45% of both genomes (Duplessis *et al.*, 2011), and *P. striiformis*, in which the TEs represent 17.8% of the generated contig sequences (Cantu *et al.*, 2011). TEs were also identified in studies of *P. pachyrhizi* studies (Posada-Buitrago and Frederick, 2005; Tremblay *et al.*, 2009; Stone *et al.*, 2012; Link *et al.*, 2014); however, in the present analysis, we provide an overall classification of the active TEs in the transcriptome of this fungus for the first time, including the identification of superfamilies in each class. We discovered a total of 592 *P. pachyrhizi* sequences with TEs, representing 1.63% of the entire transcriptome. Additionally, among the identified TEs, the majority (81.76%) were retrotransposons. Many of the superfamilies identified among TE classes have been previously identified in the rust fungus *P. striiformis* genome (Cantu *et al.*, 2011), such as the transposon superfamilies Tc1-Mariner, PIF-Harbinger, EnSpm, hAT, MuDR, P and Helitron, and the retrotransposon superfamilies Tad1, Copia, and Gypsy, of which the latter two were the most representative superfamilies in our results (61.7% of all transposable elements) and are very common in other fungi (Daboussi and Capy, 2003).

TEs can interact with the genome by means of insertions, excisions, and aberrant transpositions, even causing chromosomal rearrangements. The genomic environment, therefore, becomes a source of relevant variability, especially in species with no sexual cycle (Spanu, 2012). In phytopathogenic filamentous fungal species, effector genes were identified close to regions enriched in TEs, such as dispensable chromosomes or telomeres (Orbach *et al.*, 2000; Haas *et al.*, 2009; Ma *et al.*, 2010; Balesdent *et al.*, 2013), which may result in selective advantages for these organisms and allow a rapid response to the selection of resistance genes, as observed by Raffaele *et al.* (2010) in *P. infestans*. In plants and animals, TEs are normally stabilized during growth and development processes and can be activated by stress (Grandbastien, 1998; Capy *et al.*, 2000), but little is known about the mechanisms underlying the control of the activity of these elements in the fungal genome. Some processes have been proposed to explain this regulation in fungi, such as alternative splicing (Kempken and Kück, 1996) and homology-dependent processes such as quelling and repeat-induced point mutations (Selker, 1999; Faugeron, 2000; Cogoni, 2001; Daboussi and Capy, 2003). Although not as well-known as quelling, evidence for repeat-induced point mutations has been previously observed in other fungal species such as *P. anserina* (Graia *et al.*, 2001; Bouhouche *et al.*, 2004; Arnaise *et al.*, 2008), *M. grisea*(Ikeda *et al.*, 2002),*Leptosphaeria maculans* (Idnurm and Howlett, 2003), and *Nectria haematococca* (Coleman *et al.*, 2009).

RT-qPCR has been frequently applied to validate expression levels observed for specific genes obtained by RNA-Seq. After obtaining the *M. larici-populina* transcriptome for the uredinia stage of infected poplar leaves by LCM, Hacquard *et al.* (2010) validated the exon oligo array expression profile of the 29 transcripts encoding small secreted proteins and known rust protein homologs by RT-qPCR and thus the transcriptomic approach. In *P. pachyrhizi*, Tremblay *et al.* (2013) confirmed the RNA-Seq results for the differential gene expression of seven genes (alpha-tubulin, NADH dehydrogenase, ribulose-1,5-bisphosphate carboxylase oxygenase, pectin methyl esterase, maturase-related, serine palmitoyltransferase, and 60S ribosomal protein) also using RT-qPCR. Despite some differences between the gene expressions profiles, such as the qPCR expression levels detected at time-points that were inconsistent with the mRNA-Seq findings, both types of detection were generally consistent. Our validation of the RNA-Seq expression profiles by RT-qPCR provided similar results. The observed expression patterns for the six genes selected from the RNA-Seq data were very similar to those obtained for the RT-qPCR analyses, in which five of the six analyzed genes were induced.

The results presented in this study enrich our knowledge about the *P. pachyrhizi* transcriptome, corroborating the molecular mechanisms that have been identified in previous studies and providing new perspectives on processes that remain unknown in this pathogen. Whole-genome sequencing of this fungus as well as the functional characterization of genes related to pathogenicity during the soybean infection process are extremely important, contributing even more to the search for molecular mechanisms that may aid in the control of the disease.

## Supplementary material

The following online material is available for this article:

Table S1 - Sequences of RT-qPCR primers, amplicon size, and primer efficiency.

Table S2 - The 50 top expressed *P. pachyrhizi* transcripts at 10 days post soybean infection.
