# Supplementary material for: New insights into Phakopsora pachyrhizi infection based on transcriptome analysis in planta
Source: Genet Mol Biol. 2018 Jul-Sep;41(3):671–91. doi: 10.1590/1678-4685-GMB-2017-0161 (PMC6136362; doi:10.1590/1678-4685-GMB-2017-0161)
Supplement: Supplementary file 1 [file 1415-4757-GMB-41-03-2017-0161-20180622-suppl1.pdf]

Supplementary Material to "New insights into *Phakopsora pachyrhizi* infection based on transcriptome analysis *in planta*"

Table S1 - Sequences of RT-qPCR primers, amplicon size, and primer efficiency of selected *P. pachyrhizi* genes.

Contigs	Primers (F - forward, R - reverse)	Amplicon size (bp)	Primer efficiency (%)
de_novo_595<br>(Thi – thiamine biosynthesis)	F - TCACTGAGCTAATCGGTACAGG<br>R - GCCTTTCCAGCCAAAGTATG	73	97
de_novo_4668<br>(PPI – peptidyl-prolil-cys/trans isomerase)	F - TCGTCAGCTTTGCCTTAGAC<br>R - CTCCCTGGAGCATAAATTGG	86	98
de_novo_939<br>(AGO – argonaut)	F - ACTCGCTCGGTTTCTATTGC<br>R - CCGTTGAAATCGACGTTACC	92	90
de_novo_2740<br>(Pv_SNARE – soluble NSF attachment receptor)	F - CCGTCAATGATCCATACGTG<br>R - AGAAGTGATGCCCGTTGTTC	113	90
de_novo_5382<br>(NR – nitrate reductase)	F - CCAAGTCAAAGGTCCCAAAG<br>R - CAGCATTGGAGTTATCCCTGTC	97	98
de_novo_57<br>(HSS – small heat shock)	F - GACGCAAGTTTGATGACTCG<br>R - AACAGAGATAACCGGCTTGC	92	90
de_novo_380<br>(Tub– tubulin)	F - CCAAGGCTTCTTCGTGTTTCA<br>R - AGAGAAGAGCGCCAAACC	65	93
