# Supplementary material for: New insights into Phakopsora pachyrhizi infection based on transcriptome analysis in planta
Source: Genet Mol Biol. 2018 Jul-Sep;41(3):671–91. doi: 10.1590/1678-4685-GMB-2017-0161 (PMC6136362; doi:10.1590/1678-4685-GMB-2017-0161)
Supplement: Supplementary file 2 [file 1415-4757-GMB-41-03-2017-0161-20180622-suppl2.pdf]

Supplementary Material to "New insights into *Phakopsora pachyrhizi* infection based on transcriptome analysis *in planta*"

Table S2 - The 50 top expressed *P. pachyrhizi* transcripts at 10 days post soybean infection, based on the FPKM values, identified for each soybean genotypes PI561356 and BRS 231.

Contig	Annotation against NCBI database	FPKM values	
		BRS 231	PI561356
<u>*Common to both genotypes*</u>			
de_novo_11027	*P. pachyrhizi* clone JGIAFNA-33E19	98,342.72	48,807.68
de_novo_23913	*M. larici-populina* secreted protein with DUF3129 domain	19,815.81	29,193.11
de_novo_23959	*M. larici-populina* secreted protein with DUF3129 domain	20,080.94	27,905.95
de_novo_9895	*M. larici-populina* secreted protein with DUF3129 domain	15,173.93	21,918.25
de_novo_10151	*M. larici-populina* secreted protein with DUF3129 domain	13,719.74	18,582.40
de_novo_5849	*M. larici-populina* secreted protein with DUF3129 domain	11,467.69	16,627.02
de_novo_33003	Protein of unknown function with DUF3129 domain	11,039.74	15,825.92
de_novo_32708	No hits found	8,469.59	14,466.77
de_novo_4830	*P. pachyrhizi* clone JGIAFNA-13C12	8,744.68	13,596.40
de_novo_12325	*M. larici-populina* hypothetical protein with DUF3129 domain	9,392.71	13,079.84
de_novo_40849	No hits found	7,541.34	13,034.33
de_novo_9851	*M. larici-populina* secreted protein with DUF3129 domain	8,353.40	12,345.03
de_novo_7016	Conserved hypothetical protein with MAEBL domain	7,907.53	12,282.19
de_novo_3845	No hits found	7,849.31	12,225.83
de_novo_2238	No hits found	8,845.75	12,136.21
de_novo_5381	*M. larici-populina* secreted protein with DUF3129 domain	8,544.68	11,635.99
de_novo_6669	No hits found	5,936.03	11,509.40
de_novo_3100	No hits found	7,480.70	11,489.08
de_novo_3210	No hits found	7,388.56	11,382.46
de_novo_1402	No hits found	7,457.41	10,509.42
de_novo_3449	No hits found	5,986.86	10,209.79
de_novo_2152	No hits found	6,538.66	10,157.06
de_novo_2142	No hits found	6,573.82	10,099.46
de_novo_1770	No hits found	6,106.69	9,494.88
de_novo_2721	*P. pachyrhizi* clone JGIAFNA-5A11	5,945.71	9,249.17
de_novo_6942	No hits found	3,659.44	9,162.98
de_novo_6949	Conserved hypothetical protein with MAEBL domain	6,131.29	9,062.17
de_novo_2592	No hits found	5,847.87	9,060.92
de_novo_1478	No hits found	5,834.43	8,954.22
de_novo_8907	*P. pachyrhizi* clone JGIAFNA-33E19	1,9014.19	7,701.48
de_novo_2285	No hits found	3,954.02	7,675.55
de_novo_555	No hits found	4,633.16	7,167.71
de_novo_5657	Conserved hypothetical protein with MAEBL domain	4,650.95	6,833.45
de_novo_2037	No hits found	4,287.27	6,671.67

Contig	Annotation against NCBI database	FPKM values	
		BRS 231	PI561356
de_novo_38206	No hits found	4,250.22	6,600.62
de_novo_8211	DNA polymerase III subunits gamma and tau domain	6,113.70	6,181.58
de_novo_6732	*M. larici-populina* secreted protein with DUF3129 domain	3,837.32	5,672.38
de_novo_6277	Conserved hypothetical protein with MAEBL domain	3,730.86	5,508.55
de_novo_11704	Conserved hypothetical protein with MAEBL domain	3,733.27	5,302.30
de_novo_1254	No hits found	5,021.69	4,837.57
de_novo_1491	RNA polymerase I-associated factor PAF67	4,196.92	4,833.46
de_novo_9082	*P. pachyrhizi* clone JGIAFNA-33E19	7,804.53	4,547.20
Only for PI561356			
de_novo_13814	Galactose-3-O-sulfotransferase family	440.91	5,945.22
de_novo_4216	No hits found	3,111.34	4,925.88
de_novo_2312	No hits found	3,224.50	4,869.87
de_novo_9222	Membrane protein TolA domain (cell envelope biogenesis, outer membrane)	3,376.32	5,091.02
de_novo_6032	Protein of unknown function with DUF3129 domain	3,230.27	4,744.18
de_novo_1182	*M. larici-populina* hypothetical protein with SH2 domain	3,121.50	4,739.35
de_novo_3323	No hits found	3,020.47	4,637.93
de_novo_947	No hits found	3,454.19	4,616.23
Only for BRS 231			
de_novo_18573	Fibronectin-attachment protein domain	6,047.65	4,435.17
de_novo_3301	No hits found	5,827.95	2,238.76
de_novo_7858	No hits found	4,585.47	3,478.69
de_novo_2057	No hits found	4,390.65	3,960.86
de_novo_7893	Large tegument protein UL36 domain	3,995.37	3,909.36
de_novo_6455	DNA polymerase III subunits gamma and tau domain	3,965.85	2,812.26
de_novo_5057	No hits found	3,755.03	3,636.51
de_novo_7366	No hits found	3,751.33	3,755.50
